# Ruptured dermoid cyst of the lateral cavernous sinus wall with temporary symptoms: a case report

**DOI:** 10.1186/s13256-016-1007-3

**Published:** 2016-08-12

**Authors:** Yasushi Kosuge, Hidetaka Onodera, Taigen Sase, Masashi Uchida, Hiroshi Takasuna, Hidemichi Ito, Kotaro Oshio, Yuichiro Tanaka

**Affiliations:** Department of Neurosurgery, St. Marianna University School of Medicine, 2-16-1 Sugao, Miyamae, Kawasaki Japan

**Keywords:** Cavernous sinus, Dermoid cyst, Headache, Rupture

## Abstract

**Background:**

Dermoid cysts are non-neoplastic tumors that arise from defects in the separation of the neuroectoderm. Cyst rupture rarely occurs spontaneously and the most common symptom is headache, followed by seizure. Although many cases of ruptured dermoid cysts present with symptoms, reports of cases that are asymptomatic, or where symptoms disappear, are rare.

**Case presentation:**

We report the case of a 66-year-old Asian man with a history of sudden onset headache who was found to have high amounts of fat material in the subarachnoid space and a fat suppression mass in the left cavernous sinus. He underwent oral steroid therapy. Five days after starting medication his headache symptoms disappeared. Routine neurological imaging was then performed without surgical procedure. Magnetic resonance imaging revealed evidence of the remains of a static lesion 6 months after his first visit. He has remained headache free for 10 months since the initial event.

**Conclusions:**

Although cases of ruptured dermoid cysts presenting with consistent symptoms have been commonly reported, until now there were few reports on asymptomatic cases or cases where symptoms disappeared. We believe that surgical intervention is unnecessary for ruptured dermoid cysts with minimal symptoms.

## Background

Dermoid cysts are rare tumors, accounting for 0.04–0.6 % of all intracranial tumors that arise from ectodermally committed cells at the time of closure of the neural groove between the third and fifth week of embryonic life [1, 2]. They frequently occur at suprasellar, frontobasal, temporobasal regions, and in the posterior fossa [3]. They are benign, slow-growing tumors which rarely rupture. Dermoid cysts produce various symptoms, especially headache, seizure, cerebral ischemia, and meningitis [3, 4]. Although rupture of dermoid cysts typically occurs spontaneously, there are a few cases of traumatic rupture of dermoid cysts [5, 6]. Surgery is recommended in patients with symptomatic ruptured dermoid cysts. On the other hand, asymptomatic patients with ruptured dermoid cyst lesions are recommended for close observation, but to the best of our knowledge only eight cases with such observation have been described [3, 7–13]. We discuss the clinical features of ruptured dermoid cysts and analyze cases without surgery.

## Case presentation

**Fig. 1:**
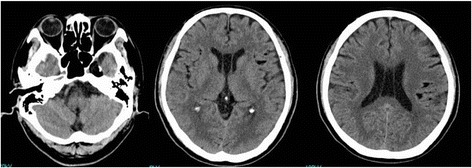
Axial unenhanced computed tomography scans showing low-density lesions in the left cavernous sinus and subarachnoid space

**Fig. 2:**
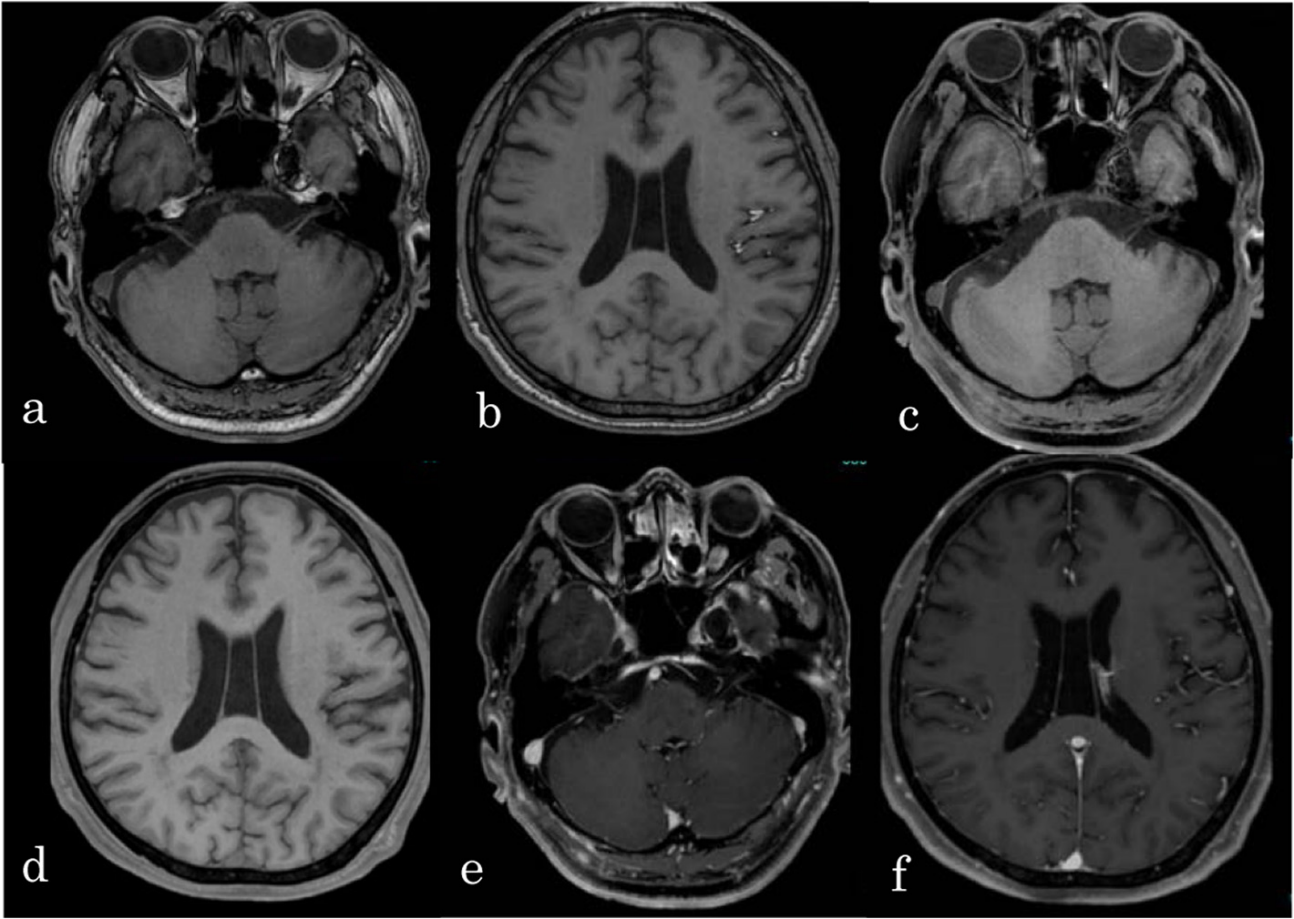
Initial magnetic resonance images of the brain. **a, b** T1-weighted images showing a 20 mm mass in the left cavernous sinus and fat drops in the Sylvian fissure. **c, d** T1-weighted fat-saturated images showing the suppression of the hyperintense drops. **e, f** T1-weighted contrast-enhanced images showing the lesions were not enhanced with gadolinium infusion

A 66-year-old Asian man presented with sudden onset headache. His past medical history was significant for intraductal papillary mucinous neoplasm. On physical examination, he was awake, alert, and his cranial nerves were intact. No neck stiffness was present. Laboratory tests were all within normal limits. A computed tomography (CT) scan of his head showed a hypodense lesion in the left cavernous sinus and many scattered fat density masses in the bilateral subarachnoid space (Fig. 1). A magnetic resonance imaging (MRI) scan of his brain revealed a 20 mm fat suppression mass and scattered small hyperintense lesions on T1-weighted image. The lesions were not enhanced with gadolinium infusion (Fig. 2). Radiographic diagnosis was ruptured cavernous sinus dermoid cyst. Oral steroid therapy with 30 mg prednisolone resulted in headache resolution 5 days later, so we decided to observe the patient with neurological imaging. The steroid was administrated for 28 days. He remained free of headache and convulsions. He did not develop double vision over the course of the follow-up. An MRI scan performed 6 months later revealed the parasellar mass and many fat droplets in the subarachnoid space unchanged since the initial MRI. At 10 months after the initial event, there was complete cessation of the headache and no seizures.

## Discussion

Dermoid cystic tumors arise from defects in the separation of the neuroectoderm at the time of neural tube closure during the third to fifth week of embryonic development [1, 2]. The cysts contain thick yellowish material consisting of sebaceous gland secretions and desquamated epithelium, in which a variable number of hairs is entangled [4]. Rupture of intracranial dermoid cysts is rare. Cyst rupture usually occurs spontaneously. A few cases of rupture after head trauma have been reported [5, 6]. Although the exact mechanism of cyst rupture remains unknown, Stendel *et al.* hypothesized that glandular secretions, possibly increased by age-dependent hormonal changes, may lead to rapid enlargement and rupture [2]. The most common symptom was headache, occurring in 31.8–32.6 % of the cases, followed by seizure in 26.5–29.5 %, and cerebral ischemia with sensory and/or motor hemisyndrome in 15.9–16.3 % [2, 4]. Ruptured dermoid cyst with chemical meningitis has been reported in 7–8.2 % of the cases, with psychosyndrome and visual disturbance present in 4.5 % [2, 4, 14].

The location of dermoid cysts were reported to be frontal base, frontal lobe, suprasellar, parasellar, Sylvian fissure, middle base, temporal lobe, hypothalamus, pineal, posterior fossa, fourth ventricle, cerebellopontine angle, and clivus [15]. They are most often found in a sellar or parasellar location as well as the frontonasal region and frequently reside near the skull base [16]. In our case, the dermoid cyst was located in the lateral wall of the left cavernous sinus. Dermoid cysts in the cavernous sinus are rare and only ten cases have been reported [1, 17–25]. The past cases of dermoid cysts in cavernous sinus presented various symptoms, such as diplopia in six cases, headache in five cases, blurring of vision in three cases, and seizure in one case. On neurological examination, third, fourth, fifth, and sixth nerve palsy were documented. In cases which were referred to therapeutic strategy, all patients underwent surgical resection. Diplopia was usually improved in cases with gross total removal. On the other hand, Akdemir *et al*. reported a case with no remarkable change in ophthalmoplegia [18]. We have an interest in dermoid cysts of the cavernous sinus and observe that there is not always a resolution of symptoms after surgery, and that patients may become asymptomatic after oral steroid therapy due to the anti-inflammatory effect.

In the case reported by Liu *et al*., surgical resection is recommended in patients with symptomatic ruptured intracranial dermoid cyst [3]. Although most cases resolve after surgery, there was relapse of symptoms and incidents of re-rupture in patients who underwent partial resection [15, 22, 26]. In cases without surgical resection, Liu *et al*. reported that close observation would be an acceptable option in these patients, although treatment of patients with asymptomatic lesions is not yet well codified [3]. The number of reports that evaluate observation with regular neurological imaging is small and the natural history of ruptured dermoid cysts is uncertain. To the best of our knowledge only nine patients including our case have been reported to be treated conservatively. We reviewed patients who were subjected to conservative management (Table 1) [7–13]. Ruptured cysts were located in parasellar and suprasellar regions except for one case at the pineal region. Asymptomatic lesions accounted for only two cases, with other cases all having had initial symptoms. Neuroradiological findings were characterized by a lack of change in fat droplets of the subarachnoid space over the course of conservative management. Many reported cases which were treated by surgery presented with visual disturbance. On the other hand, there is no example of a report with visual disturbance in conservatively managed cases. In closely observed cases, the initial symptoms of ruptured dermoid cyst, including headache, seizure, meningitis and paresis, were present (with two asymptomatic exceptions), but most cases were uneventful and asymptomatic over the course of observation, with the exception of one with seizure. Headache is believed to be caused by the mass effect of the cyst as well as due to meningeal irritation caused by the rupture of the dermoid cyst [2]. In our case, the dermoid cysts were too small to produce mass effects, so the oral steroid therapy which made the headaches disappear presumably decreased the meningeal irritation. A separate case in which steroid treatment was equally effective has been reported by Wang *et al*. [12]. Therefore, we consider conservative treatment to be an approach which should be examined as a therapeutic alternative in cases with headache or controllable seizure without visual disturbance. But, the course of observation for ruptured intracranial dermoid cysts is so far largely anecdotal and unclear in the absence of a large series of long-term follow-up cases. We conclude that it is important for long-term follow-up cases of ruptured dermoid cyst to be systematically accumulated.

**Table 1:** Literature review of the ruptured dermoid cyst with conservative management

Case	Age (years)	Sex	Location	Initial symptoms	Follow-up
Wilms *et al*. [13]	16	F	Parasellar	Seizure, paresis, aphasia	6 years
Corr *et al*. [7]	NA	F	Pineal	Headache, meningitis	NA
Messori *et al*. [10]	NA	M	Parasellar	Asymptomatic	2 months
Messori *et al*. [10]	22	F	Parasellar	Asymptomatic	3 years
Rajapakse *et al*. [11]	76	F	NA	Headache	NA
Kucera *et al.* [9]	19	M	Suprasellar	Seizure, meningitis	10 months
Indullar *et al*. [8]	17	F	Suprasellar	Headache, meningitis	2 years
Wang *et al*. [12]	71	M	NA	Headache, meningitis	1 week
Our case	66	M	Parasellar	Headache	10 months

*NA* not applicable, *M* male, *F* female

## Conclusions

Ruptured dermoid cysts presenting with asymptomatic or disappearing symptoms are rare. The number of reports that evaluate observation with regular neurological imaging is small and the natural history of ruptured dermoid cysts is uncertain. We believe conservative treatment without surgery should be considered a therapeutic alternative in asymptomatic cases of ruptured dermoid cyst, or where symptoms disappear.
